# The Evolution of Polymer Composition during PHA Accumulation: The Significance of Reducing Equivalents

**DOI:** 10.3390/bioengineering4010020

**Published:** 2017-03-07

**Authors:** Liliana Montano-Herrera, Bronwyn Laycock, Alan Werker, Steven Pratt

**Affiliations:** 1 School of Chemical Engineering, University of Queensland, St Lucia QLD 4072, Australia; liliana.montano@usys.ethz.ch (L.M.-H.); b.laycock@uq.edu.au (B.L.); 2 Veolia Water Technologies AB—AnoxKaldnes, Klosterängsvägen 11A SE-226 47 Lund, Sweden; alan@werker.se

**Keywords:** PHA, monomer evolution, mixed culture, modeling, polymer composition, biopolymer

## Abstract

This paper presents a systematic investigation into monomer development during mixed culture Polyhydroxyalkanoates (PHA) accumulation involving concurrent active biomass growth and polymer storage. A series of mixed culture PHA accumulation experiments, using several different substrate-feeding strategies, was carried out. The feedstock comprised volatile fatty acids, which were applied as single carbon sources, as mixtures, or in series, using a fed-batch feed-on-demand controlled bioprocess. A dynamic trend in active biomass growth as well as polymer composition was observed. The observations were consistent over replicate accumulations. Metabolic flux analysis (MFA) was used to investigate metabolic activity through time. It was concluded that carbon flux, and consequently copolymer composition, could be linked with how reducing equivalents are generated.

## 1. Introduction

Polyhydroxyalkanoates (PHAs) are biobased and biodegradable polyesters. PHA copolymers, such as poly(3-hydroxybutyrate-*co*-3-hydroxyvalerate) (PHBV), are of particular interest as they are the basis for biomaterials with desirable mechanical properties. These copolymers can be produced in mixed microbial cultures [1]. However, predicting and controlling the copolymer composition can be challenging.

PHAs are most typically synthesized in mixed microbial cultures from volatile fatty acids (VFAs), through well-described metabolic pathways [2,3]. In the specific case of PHBV, short chain acids such as acetic and propionic acids are transported though the cell membrane and converted into acetyl-CoA and propionyl-CoA respectively. PHA synthesis then takes place in three steps. Firstly, two acyl-CoA molecules are condensed in a reaction catalyzed by a thiolase to produce various intermediates. For example, two acetyl-CoA monomers form acetoacetyl-CoA (a 3-hydroxybutyrate (3HB) precursor), while one acetyl-CoA and one propionyl-CoA combine to form ketovaleryl-CoA (a 3-hydroxyvalerate (3HV) precursor) [4], Escapa et al. 2012). In addition, it has been observed that a portion of the propionyl-CoA produced is converted into acetyl-CoA though different pathways [5]. Secondly, a reduction, catalyzed by a reductase, produces 3-hydroxyalkanoate (3HA) monomers, with the reducing power to support PHA production being generated during anabolic pathways for cell growth, as well as in reactions related to the tricarboxylic acid (TCA) cycle [6]. Finally, a polymerase adds 3HA monomers to the PHA polymer. As such, the flux of carbon through the acyl-CoA intermediates influences the resulting polymer composition.

The fraction of 3HV monomer units in the final PHBV copolymer can be manipulated by adjusting the proportion of even-chain (i.e., acetic acid) to odd-chain (i.e., propionic acid) fatty acids in the feed composition [7,8,9], since odd-chain fatty acids are generally required for the formation of propionyl-CoA, which is the precursor of 3HV monomer. Diverse monomer compositions and sequence distributions of PHBV copolymers produced by mixed microbial cultures have been achieved using different feeding strategies with acetic and propionic acid mixtures as model substrates [10,11].

Most mixed microbial culture accumulation studies have been applied under conditions of some form of nutrient starvation to inhibit cell growth and favor PHA synthesis [12,13,14]. In contrast, a recent study has shown that PHA storage can occur concurrently with active biomass growth. Valentino et al. [15] achieved a consistent improvement of PHA productivity when N and P were supplied in an optimal C:N:P ratio. It is important to consider that shifts in the active biomass growth rates may influence carbon flux through the acyl-CoA intermediates and the availability of reducing equivalents for PHA synthesis, and therefore affect polymer production and composition.

Literature on mixed microbial culture PHA production coupled with high rate cell growth is scant. Simulations of existing models have successfully fitted data on PHA productivity and even monomer composition evolution in some cases [4,16,17]; however, these models apply only for scenarios of negligible growth. In addition, in these experiments the feedstock composition was kept constant during the accumulation process resulting in a polymer with a constant ratio of 3HB:3HV. These existing model frameworks are in contrast to some published experimental data that do show shifts in copolymer composition during fed-batch mixed culture PHA accumulation, even under non-growing conditions [11]. Such data indicate that the 3HB:3HV ratio during accumulation is not simply dependent on the feedstock but is also affected by the history of the accumulation and the resulting metabolic activity in the biomass. The potential for biomass growth and other processes to directly influence the composition of intracellular acyl-CoA reservoirs and hence copolymer composition has not been examined.

The aim of this paper is to examine 3HB and 3HV monomer evolution through PHA accumulation, giving consideration to the effect of biomass growth and alternating feedstocks on this process. To this end, monomer development through four sets of PHA accumulation experiments (based on the feeding regime) is investigated: Set 1: acetic acid (HAc) feed; Set 2: propionic acid (HPr) feed; Set 3: mixed HAc and HPr feed; and Set 4: alternating HAc/HPr feed. Concurrent biomass growth and carbon storage is encouraged in each set. Metabolic Flux Analysis (MFA) is used to quantify metabolic pathway activity through the accumulations.

## 2. Materials and Methods

### 2.1. Experimental Set-Up

PHA was produced at pilot scale at AnoxKaldnes AB (Lund, Sweden) using a three stage process that had been in continuous operation from 2008 to 2013 [11]. The first stage (acidogenic fermentation) was performed in a 200 L continuous stirred tank reactor under anaerobic conditions and fed with cheese whey permeate, producing a mixture comprising 35% ± 4% acetic, 4% ± 1% propionic, 49% ± 4% butyric, 4% ± 1% valeric and 8% ± 3% caproic acids. The second stage was carried out in a sequential batch reactor (SBR) operated under Aerobic Dynamic Feeding (ADF) conditions with nutrient addition (COD:N of 200:5). The excess biomass with a high PHA storage capacity (as enriched in stage two) was used to produce PHA-rich biomass in the third stage, in a reactor operated in fed batch mode. The details of this process and analytical methods can be found in Janarthanan et al. [18].

### 2.2. Fed-Batch PHA Production

PHA was accumulated in batches of 100 L harvested SBR mixed liquor by means of a 150 L (working volume) aerated reactor. Aeration provided mixing as well as oxygen supply. Acetic and propionic acids (HAc and HPr, respectively) were fed using different HAc:HPr ratios and feeding strategies. The microbial community was dominated by the genera *Flavisolibacter* and *Zoogloea* [18].

The carbon source concentration for pulse-wise substrate addition was ~100 gCOD/L (see Table 1), with pH adjusted to 4 and additions of nitrogen and phosphorus for nutrient limitation to give COD:N:P of 200:2:1 [15]. N and P additions were 3.82 g/L NH_4_Cl and 0.22 g/L KH_2_PO_4_, respectively. For the fed-batch accumulations, a pulse-wise feedstock addition was applied for feed-on-demand [19] controlled by the biomass respiration response as measured by dissolved oxygen (DO) trends [7,11]. Semi-continuous (pulse-wise) additions of feedstock aliquots were made targeting peak COD concentrations of between 100 and 200 mg-COD/L. Feedstock additions were triggered by a measured relative decrease in biomass respiration rate [7]. pH was monitored but not controlled. The fed-batch accumulations were run over 20–25 h with samples taken at selected times for analyses, including VSS (volatile suspended solids), TSS (total suspended solids), PHA content and composition, soluble COD, volatile fatty acids (VFAs), and nutrients (nitrogen and phosphorus).

### 2.3. Analytical Methods

Total concentrations were analyzed from well-mixed grab samples and soluble concentrations were analyzed after filtering the aqueous samples with 1.6 μm pore size (Ahlstrom Munktell, Falun, Sweden) filters. Volatile fatty acid concentrations were quantified by gas chromatography [20]. Solids analyses (total and volatile suspended solids, or TSS/VSS) were performed according to Standard Methods [21].

Hach Lange™ kits were used for the determination of soluble COD (sCOD) (LCK 114), NH_4_–N (LCK 303), NO_3_–N (LCK 339), soluble total phosphorus (LCK 349) and soluble total nitrogen (LCK 138). PHA content and monomeric composition (3HB and 3HV) of samples was determined using the gas chromatography method described in [11] using a Perkin-Elmer gas chromatograph (GC) (Perkin Elmer, Inc., Waltham, MA, USA). Quantitative ^13^C high resolution NMR spectra were acquired on a Bruker Avance 500 spectrometer (Bruker, Billerica, MA, USA) as described by Arcos-Hernandez et al. [11] to determine polymer microstructure (details of polymer structure can be found in Supplementary Materials).

### 2.4. Experimental Design for PHA Accumulations

The full set of PHA accumulation (third stage) experiments are summarized in Table 1. For this work, four experiments (replicated, with replicates denoted using the symbol ’) were considered. Experiment set 1 used a single acetic acid (HAc) substrate; Experiment set 2 used a single propionic acid (HPr) substrate; Experiment set 3 used a mixed HAc and HPr substrate fed simultaneously in equal COD ratios; while in Experiment set 4 the acids were supplied in alternating pulses.

### 2.5. Rate and Yield Calculations

For PHA concentration at a given time (in *g PHA*/*L*), only the PHA produced during the accumulation process was considered. Therefore, the measured PHA concentration (*PHA*) was corrected by subtracting the initial measured PHA content (*PHA*_0_). Typically the initial biomass PHA content (%*PHA*_0_ in wt %) was between 0% and 4%. Active biomass (CH_1.4_O_0.4_N_0.2_) at a given time (*X*, recorded in g/L) was determined from the total concentration of biomass, measured as volatile suspended solids (*VSS* in *g VSS*/*L*), subtracting the produced PHA concentration (*PHA*):

X= VSS (g VSS/L) − PHA (g PHA/L)

*PHA* intracellular content (%*PHA*) was calculated as the PHA concentration divided by the volatile suspended solids concentrations on a mass basis.

$$\%PHA\;\left(gPHA/g\;VSS\right)\;=\frac{PHA\;\left(g\;PHA/L\right)}{VSS\;\left(g\;VSS/L\right)}$$

The PHA fraction ($f_{PHA})\;$was measured as PHA concentration divided by active biomass concentration on a COD basis.

$$f_{PHA}=\frac{PHA\;\left(in\;gCOD\;PHA/L\right)}{X\;\left(in\;mCOD\;X/L\right)}$$

Experimental data for the total amount of VFA consumed, PHA polymer (*PHA*) produced and active biomass (*X*) produced were fitted using global nonlinear regression in GraphPad Prism (v.6.0.5). This analysis was performed using an exponential growth model (one phase association) [22]. The batch process mass balance accounted for input feed dosing volumes as well as sampling withdrawal volumes. Kinetic rates and yields were calculated from fitted data as follows:

Acetic ($q_{HAc})$ and propionic acid $\left(q_{HPr}\right)$ specific consumption rates and specific monomer 3HB and 3HV production rates: $q_{HB}$ and $q_{HV}$, respectively, for the *i*th uptake of each acid or production of each monomer were calculated with reference to active biomass (*X*) concentration:

$$q_{HAc}=\frac{\left(HAc_{i}-HAc_{i-1}\right)}{\left(t_{i}-t_{i-1}\right)\cdot X_{i}}\;q_{HPr}=\frac{\left(HPr_{i}-HPr_{i-1}\right)}{\left(t_{i}-t_{i-1}\right)\cdot X_{i}}$$

$$q_{S}\;\left(Cmol\;VFA\cdot Cmol\;X^{-1}\cdot h^{-1}\right)=q_{HAc}+q_{HPr}$$

$$q_{HB}=\frac{\left(3HB_{i}-3HB_{i-1}\right)}{\left(t_{i}-t_{i-1}\right)\cdot X_{i}}\;q_{HV}=\frac{\left(3HV_{i}-3HV_{i-1}\right)}{\left(t_{i}-t_{i-1}\right)\cdot X_{i}}$$

$$q_{PHA\;}\left(Cmol\;PHA\cdot Cmol\;X^{-1}\cdot h^{-1}\right)=q_{HB}+q_{HV}$$

where *t* is time; *HAc* and *HPr* are the moles of acetic and propionic acids in solution; *3HB* and *3HV* are the moles of 3HB and 3HV, respectively; and $q_{S}$ is the specific consumption rate of substrate (S). The instantaneous relative rate change in 3HV monomers ($\%3HV^{inst}$) was calculated relative to the total PHA specific production rate on a mole basis.

$$\%3HV^{inst}=\;\frac{q_{HV\;}\left(mol\;3HV\cdot h^{-1}\cdot X^{-1}\right)}{q_{PHA}\;\left(mol\;PHA\cdot h^{-1}\cdot X^{-1}\right)}$$

As previously reported by Janarthanan et al. [18], a linear correlation was obtained between gCOD PHA produced versus total substrate consumed (also in gCOD) and the yield (*Y_PHA_*_/*S*_) in gCOD PHA/gCOD S at 20 h was determined (0.968 < r^2^ < 0.998). This time point was selected for consistent comparison between runs as all accumulations had reached at least 98% of plateau PHA content by this time. Likewise, plots of active biomass (in gCOD X) versus time were represented by linear regression to a linear quadratic equation, and the yield (*Y_X_*_/*S*_) in gCOD X/gCOD S at 20 h was determined. The 95% confidence intervals associated with all the determined stoichiometric and kinetic parameters were estimated using error propagation formulae. The values were also converted to Cmol basis.

Maximum specific growth rate (μ*_max_*) was calculated according to the re-parameterization of the empirical expression applied to growth curves developed by Gompertz [23]. Analysis was performed in SigmaPlot (Systat Software, v.12) plotting ln(*X*/*X*_0_) versus time (0.938 < r^2^ < 0.989).

The maximum specific VFA consumption rate (*q_S_*, Cmol VFA/(Cmol X·h)) and maximum specific PHA storage rate (*q_PHA_*, Cmol VFA/(Cmol X·h)), were determined from the trends in the experimental data during the exponential growth phase. The ratio of PHA concentration and total VFA consumed divided by the active biomass concentration at that time were plotted over time, calculating the first derivative.

### 2.6. Metabolic Flux Analysis (MFA)

**Figure 1:**
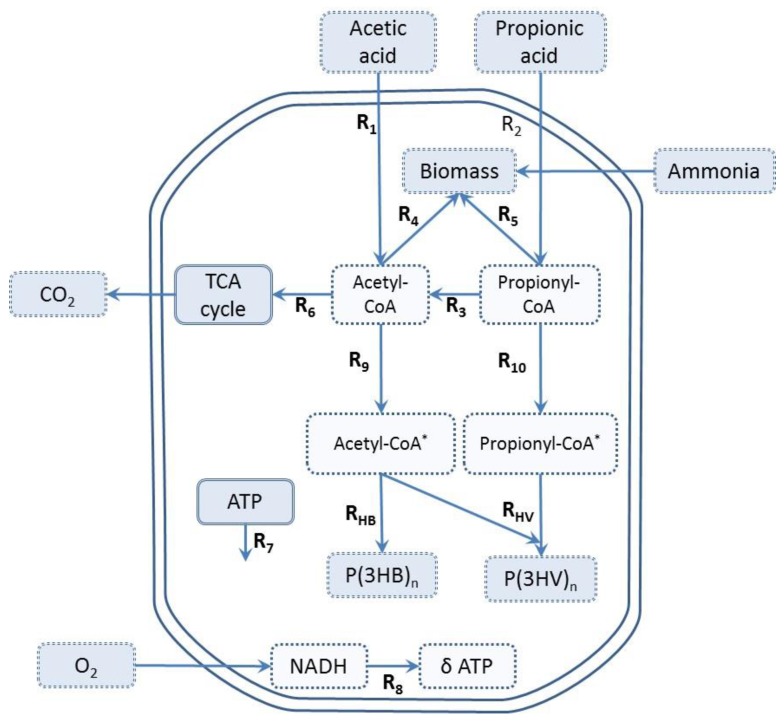
Metabolic network for PHBV synthesis and biomass production, adapted from [17], with permission from © 2013 Elsevier. Light blue dotted squares represent external metabolites; white dotted squares represent internal metabolites.

MFA was performed in order to investigate the effect of VFA composition and the feeding strategy on active biomass growth and PHA (3HV and 3HB) monomer formation kinetics assuming a pseudo-steady state. The metabolic network used in this work is based on previously published models [4,17] and summarized in Figure 1. The reactions R_9_ and R_10_ (Figure 1) describe the conversion of acetyl-CoA and propionyl-CoA into PHA precursors, where acetyl-CoA* and propionyl-CoA* are representations of molecules which have undergone the first two steps of PHA synthesis (condensation and reduction) [2,4]. Subsequently, PHA precursors are polymerized to form the biopolymer (PHB and PHV), with two units of acetyl-CoA* forming one 3HB molecule, and one unit of acetyl-CoA* and one of propionyl-CoA* forming one molecule of 3HV. The cells obtain energy from adenosine triphosphate (ATP), which is generated by the oxidation of NADH, and the efficiency of ATP production is represented by the phosphorylation efficiency (P/O) ratio (δ). The maximum theoretical P/O ratio is 3 mol-ATP/mol-NADH_2_ in bacteria growing under aerobic conditions [24].

The metabolic model consists of 12 reactions, 6 intracellular metabolites (acetyl-CoA, propionyl-CoA, acetyl-CoA*, propionyl-CoA*, ATP, and NADH), 4 substrates (HAc, HPr, O_2_, and NH_4_), and 4 end products (3HB, 3HV, X, and CO_2_). The system of equations has six degrees of freedom [25], and a total of seven rates were measured (VFA consumption rates, PHA monomers storage rate, oxygen uptake rate, active biomass synthesis rate, and ammonium consumption). Therefore, the system is overdetermined, with one degree of redundancy, which made it possible to estimate the experimental errors in measurements.

The following constraints and assumptions were set for MFA:

- Active biomass can be formed either from acetyl-CoA or propionyl-CoA. Previous models were performed under ammonia limiting conditions with negligible cellular growth [4,13]. In the present work, we assumed that fluxes of acetyl-CoA and propionyl-CoA used for active biomass ($v_{4},\;\;v_{5}$) synthesis are proportional to the consumption rate of acetic and propionic acid ($v_{HAc},\;v_{HPr}$) [16].
  $$f_{Pr}=\frac{\;v_{HPr}}{\;v_{HAc}+\;v_{HPr}}$$
  $$\frac{v_{4}}{v_{5}}=\frac{1-f_{Pr}}{f_{Pr}}$$
- Reactions R_9_ and R_10_ are reversible, the rest are irreversible reactions.
- The maintenance requirement ($v_{7}$) was an estimated flux while the P/O ratio was fixed (δ = 3).
- PHA depolymerization was not considered.

MFA was performed using the CellNetAnalyzer (v. 2014.1, Max Planck Institute, Magdeburg, Germany) toolbox for Matlab [26]. To evaluate the consistency of experimental data with the assumed biochemistry and the pseudo-steady state assumption a *chi-squares-test* was carried out. The flux distributions calculated were found to be reliable given that the consistency index (*h*) values were below a reference chi-squared test function (χ^2^ = 3.84 for a 95% confidence level and 1 degree of redundancy) [27]. The stoichiometry of the metabolic reactions is provided in the Supplementary Materials.

## 3. Results and Discussion

### 3.1. Biomass Growth and PHA Content

**Figure 2:**
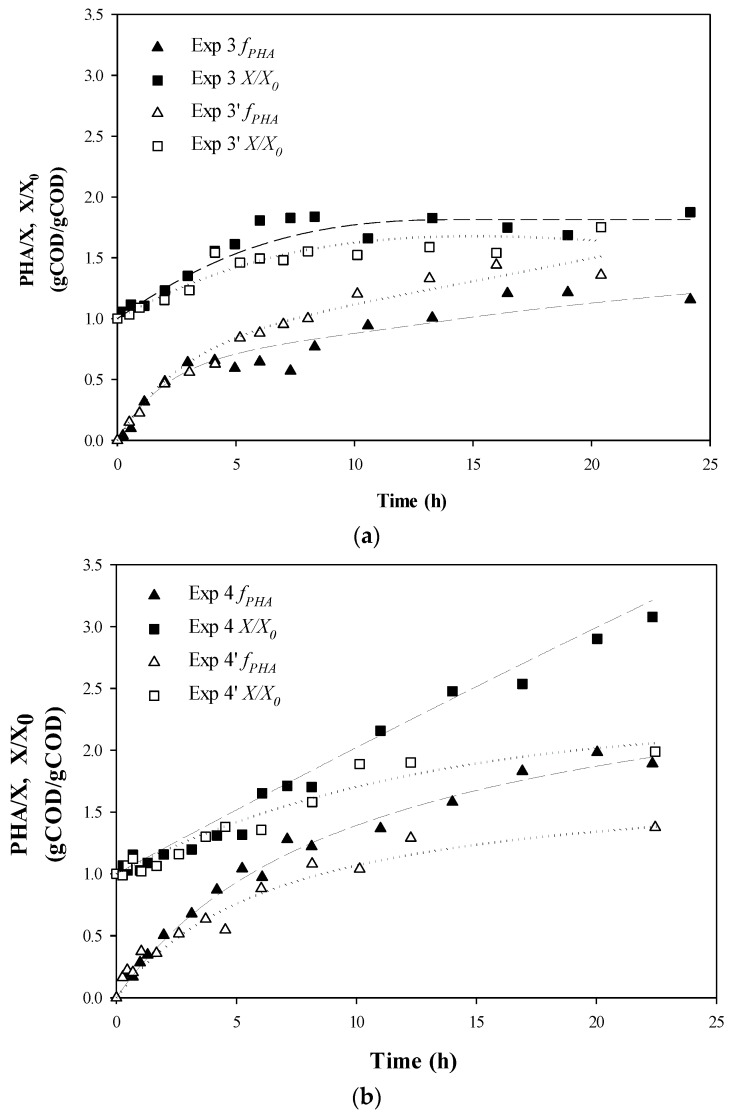
Experimental data for PHA fraction and relative active biomass production: (**a**) Experiment set 3: 50% acetic acid and 50% propionic acid fed simultaneously; and (**b**) Experiment set 4: 100% acetic acid alternating with 100% propionic acid. ▲, ∆, PHA fraction respect to active biomass concentration *f_PHA_* (*PHA*/*X*); ■, □, Active biomass concentration respect to the initial biomass concentration (*X*/*X*_0_). Dotted lines represent fitted data. Coefficients are given on gCOD basis.

The experiments were designed to follow the time evolution of PHA storage and active biomass growth during the third stage of the PHA-production system and representative Experiment sets 3 and 4 are shown in Figure 2 (sets 1 and 2 can be seen in the Supplementary Materials). The extent of production of active biomass was variable between the experiments, but higher maximum specific growth rates were achieved for accumulations where acetic acid was fed (Experiment set 1 with 100% HAc, Experiment set 3 with 50%/50% HAc/HPr, and Experiment set 4 with alternating substrates) (Table 2). However, active biomass growth rates attenuated sooner for those accumulations where acetic acid was present at all times (Experiment set 1 and Experiment set 3), while the highest biomass production (X/X_0_) was achieved in Exp 4 (Figure 2, Table 2). With regard to PHA fraction evolution, a similar PHA content at plateau was achieved for all experiments. However, PHA content and yield tended to be higher in those accumulations with alternating substrates (Experiment set 4), with one experiment (Exp 4) maintaining an increasing PHA fraction even after 22 h of accumulation. This observation fits with the interpretations from other works that it is possible to stimulate PHA storage with concurrent cellular growth by supplying an optimal nutrient ratio [6,15,18].

### 3.2. Monomer Development

**Figure 3:**
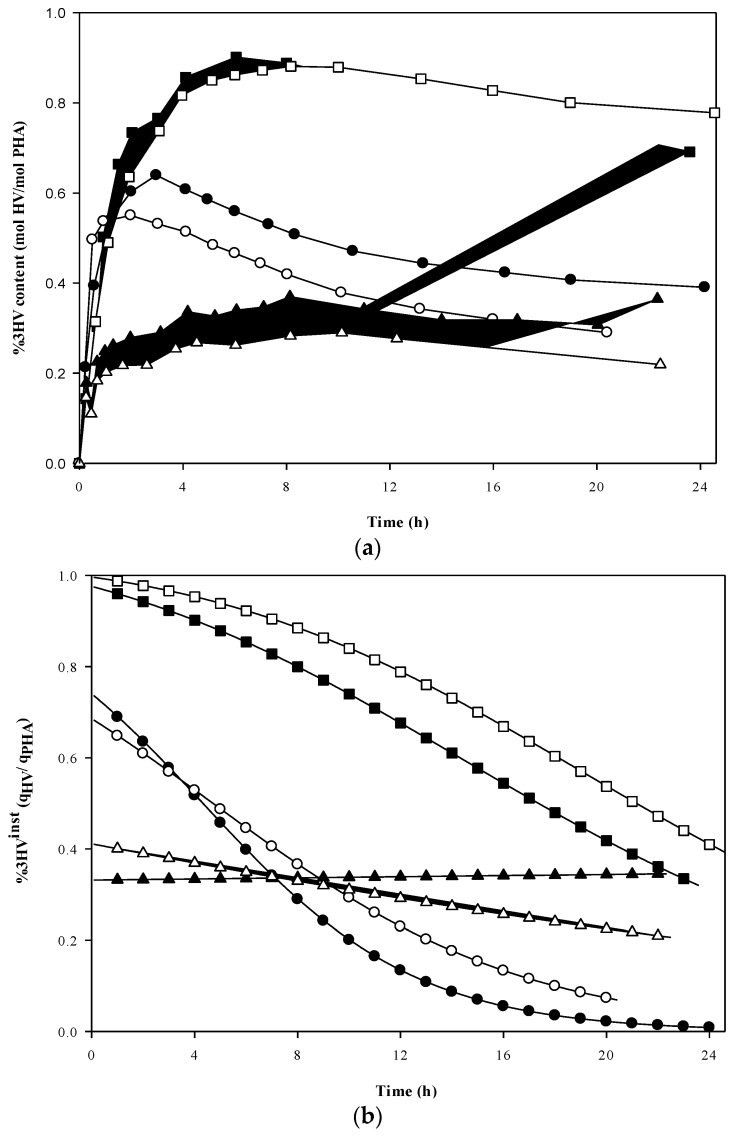
Evolution of 3HV fraction during PHA accumulation using different feeding strategies: (**a**) accumulated 3HV fraction; and (**b**) instantaneous 3HV fraction calculated from data regression. Experiment set 2: 100% propionic acid (■, Exp 2; and □, Exp 2′). Experiment set 3: 50% acetic acid and 50% propionic acid fed simultaneously (●, Exp 3; and ○, Exp 3′). Experiment set 4: 100% acetic acid alternating with 100% propionic acid (▲, Exp 4; and ∆, Exp 4′).

The polymer composition over time during the accumulations is shown in Figure 3a, while the flow of carbon to 3HV relative to PHA overall at each time point (the instantaneous 3HV fraction) is shown in Figure 3b. The trends of replicate runs all matched well with the originals in terms of monomer development, although the final 3HV content differed slightly from run to run. The highest values of 3HV content were achieved in accumulations with propionic acid present at all times; Experiment set 2 (100% HPr) and Experiment set 3 (50%/50% HAc/HPr) reached a maximum %3HV content of 0.90 and 0.72 (on a mole % basis) at 6 and 2 h, respectively (Figure 3a). Although the formation rates of 3HV units relative to the formation of PHA dominated in the early stages of the accumulation for Sets 2 and 3, a sharp decrease in the instantaneous 3HV fraction was identified (Figure 3b). In contrast, the instantaneous 3HV fraction (%3HV^inst^) in Experiment set 4, which followed an alternating pulse feeding strategy of acetic and propionic acids, did not show any remarkable change with time. However, it should be noted that %3HV^inst^ steadily decreased during Exp 4′ but gradually increased during Exp 4 (Figure 3b). The trend in %3HV^inst^ in Exp 4 coincided with high production of active biomass in that system (Figure 3a). Overall, concurrent PHA storage and active biomass growth resulted in a dynamic trend in polymer composition, except for the alternating feeding strategy.

Current models for mixed culture PHA production using acetic and propionic acids as substrates consider that the proportion of 3HV monomer units in the copolymer obtained changes in proportion to the relative composition of the carbon feeds. This assumption has often adequately predicted 3HV:3HB molar composition in cultures with negligible cellular growth. However, when a shifting substrate strategy was applied, and moreover when cellular growth was maintained, even while maintaining a constant feed composition, then the published models cannot predict the observations of the present investigation. Metabolic analysis of the carbon flux distribution through time reveals why this would be so.

### 3.3. Carbon Flux to PHA, Biomass and CO_2_

**Figure 4:**
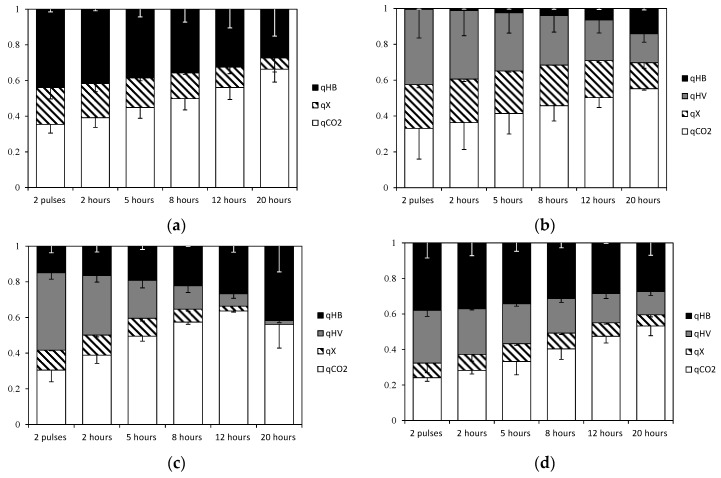
Carbon flux distribution (*q_PHB_*, *q_PHV_*, *q_X_*, *q_CO2_*) normalised respect to substrate uptake rate: (**a**) Experiment set 1: 100% acetic acid; (**b**) Experiment set 2: 100% propionic acid; (**c**) Experiment set 3: 50% acetic acid and 50% propionic acid fed simultaneously; and (**d**) Experiment set 4: 100% acetic acid alternating with 100% propionic acid.

Figure 4 shows the calculated carbon flux distribution to PHA monomers, active biomass and carbon dioxide for the four experiment sets at various time points. Of note is an increase in the proportion of carbon flux directed to carbon dioxide (CO_2_) production over time, mostly due to the carbon flux through the TCA cycle (R_6_) in all experiments, while VFAs were being consumed despite a reduction of active biomass synthesis rate and PHA production rate. According to Escapa et al. [28], the uptake of carbon source in cultures of *Pseudomonas putida* with no PHA synthase activity remains active and the excess is directed to the TCA cycle which produces CO_2_, as a way to dissipate the carbon surplus.

In the model used in the present study, it was considered that the extra carbon consumed was passed to the TCA cycle for production of high energy molecules (ATP and NADH), and dissipated as ATP (Table 3). When the VFA consumption rate exceeded the respiratory capacity, a slightly decreasing trend in the respiratory quotient was observed. It should also be noted that no pathways for polymer consumption were included, although it is known that polymerization and PHA consumption can occur simultaneously [29]. It has been demonstrated that PHA operon proteins, including PHA depolymerase, are expressed from the start of the growth phase in *Pseudomonas putida* [30]. The present model was found to be feasible, if this extra carbon either goes to a depolymerization pathway or if it is spilled to produce ATP in R_7_ which accounts for non-growth associated ATP maintenance. In both scenarios the same flux of CO_2_ is predicted. Further model improvements that measure probable depolymerization subproducts and CO_2_ production rates would be necessary to confirm the balances.

Comparing experiments with single substrates, more CO_2_ is generated when acetic acid is the only substrate compared with when propionic acid is used. The TCA cycle was calculated to be more active in Experiment set 1 (100% acetic acid), because more energy is needed to metabolize acetic acid (given 1 mol ATP is necessary to produce 1 Cmol acetyl-CoA, while activating 1 Cmol of propionic acid consumes only 0.67 mol ATP, see stoichiometry in the Supplementary Materials). On the other hand, propionic acid was found to have a higher oxygen demand when acetyl-CoA is formed from propionyl-CoA decarboxylation [31], leading to a decreased respiratory quotient (RQ). The MFA results were in agreement with this expectation. Experiment set 2, fed with propionic acid as a single substrate, had a lower RQ compared with Experiment set 1 (Table 3). However, for feeding strategies with mixed substrates, (Experiment set 3 and Experiment set 4), the RQ was very similar and remained at similar values throughout the accumulation experiments. The latter observations agreed with the composition data: similar molar fractions of propionic acid in the feed for Experiment set 3 and Experiment set 4 resulted in a constant molar fraction of propionic acid uptake relative to total carbon uptake flux (*f_Pr_*).

Concerning active biomass synthesis, reaction stoichiometry indicates that propionyl-CoA gives higher theoretical growth yields compared with acetyl-CoA (1.06 mol PrCoA produces 1 mol X, and 1.27 mol AcCoA generates 1 mol X, see Supplementary Materials). According to this MFA analysis, propionyl-CoA was diverted to cell growth and PHA production during the exponential phase growth (0–8 h) in those accumulations that had propionic acid present at all times (see Experiment sets 2 and 3 in Figure 4b,d as examples), with propionyl-CoA having been shown to be the preferred substrate for active biomass growth by Lemos et al. [32] and Jiang et al.[16]. However, at the end of all experiments, when the decarboxylation rate was high, acetyl-CoA units were converted into 3HB monomers. As a consequence, 3HV monomers dominate in the early stages of accumulation, with their formation rate decreasing over time. To understand this result, there needs to be some consideration of the role that generation of reducing equivalents plays in controlling the pathways.

### 3.4. Pathways for Generation of Reducing Power

There are three important cofactors involved in PHA synthesis and regulation: coenzyme-A, NADH/NAD^+^ and NADPH/NADP^+^ [28,33]. The relative concentrations of acyl-CoA and free coenzyme-A are critical in controlling metabolic pathways, in particular PHA storage. NADH participates in catabolic reactions, while NADPH has an important role in reductive biosynthesis such as PHA biopolymers and active biomass [33]. In reduced metabolic networks, NADPH is not considered separately. The model used in the present study, developed by Pardelha et al. [17], works under the assumption that there exists free interchange between the reducing equivalents NADPH and NADH [34].

PHA production is favored when NADPH concentrations and NADPH/NADP^+^ ratios are high [13]. In the model used in this current study, both PHA accumulation and energy production during oxidative phosphorylation require a source of reducing power. Therefore, the metabolic processes where reducing equivalents (NADH) are formed become key factors. Considering the processes outlined in Figure 1, these are reactions related to cellular growth (R_4_ and R_5_), the TCA cycle (R_6_) and decarboxylation of propionyl-CoA to acetyl-CoA (R_3_). In this sense, fluctuations in active biomass growth and PHA synthesis activities could be related to changes in the flux though the TCA cycle and/or decarboxylation of propionyl-CoA. On the other hand, VFA uptake and cell growth have an ATP requirement which is met by the TCA cycle and the electron transport of the respiratory chain [35].

One limitation for maximizing PHA yields is the regeneration of reducing equivalents (NADPH/NADH). MFA results showed that most of NADH was generated by the TCA cycle. According to the metabolic model, less reductive equivalents are necessary to produce 1 Cmol of propionyl-CoA* than 1 Cmol of acetyl-CoA* (0.167 vs. 0.25 mol NADH, respectively). Therefore, in order to produce 3HB monomer units, a greater amount of carbon must be directed to TCA cycle for NADH generation reducing equivalents (NADH).

**Figure 5:**
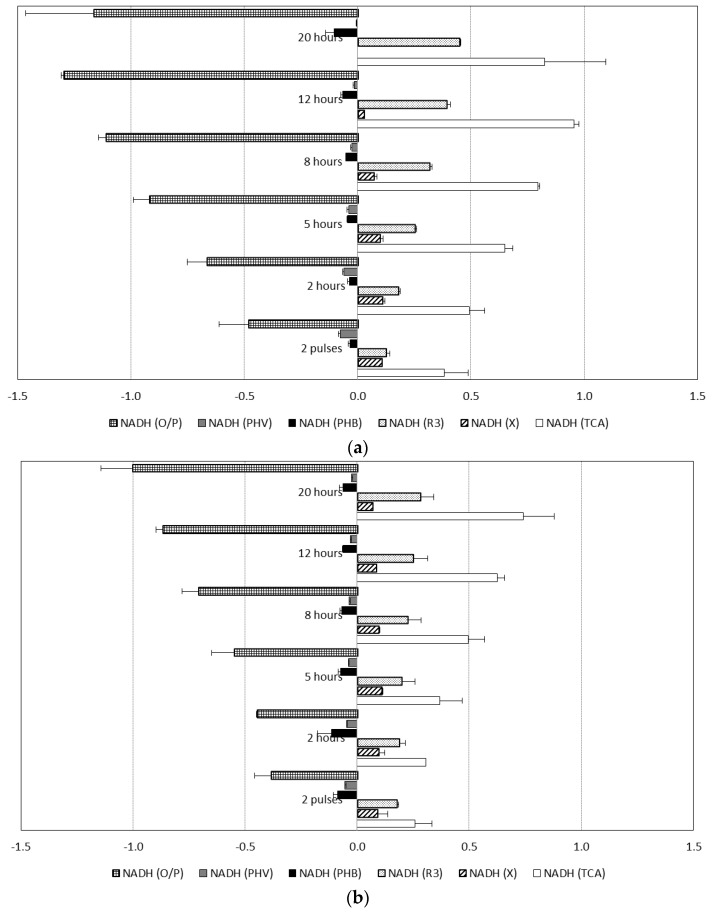
NADH generated and consumed at different stages of culture obtained by metabolic flux analysis (MFA): (**a**) Experiment set 3: 50% acetic acid and 50% propionic acid fed simultaneously; and (**b**) Experiment set 4: 100% acetic acid alternating with 100% propionic acid. (NADH was considered as internal metabolite).

To investigate the role of these pathways for generation of reducing equivalents in evolution of copolymer composition, metabolic flux analysis (MFA) was performed at different stages of the accumulations for all the feed regimes tested (Experiment sets 1, 2, 3, and 4) (see Figure 5).

In cultures fed with alternating substrates (Exp 4 and 4′), carbon fluxes for active biomass formation and 3HV monomer production remained constant during the accumulations (Figure 4). As shown in Figure 5b, NADH generation rate by propionyl-CoA decarboxylation (*v*_3_) was kept low, to a level to cover energy production by TCA cycle requirements. On the other hand, in experiments with mixed acetic and propionic feeds (Exp 3 and 3′), the decarboxylation rates increased markedly when the active biomass growth rates attenuated, and thus more acetyl-CoA units became available and the 3HB production rate could increase as a result.

As mentioned previously, propionic acid has been shown to be the preferred substrate for active biomass formation. Production of pure 3HV was not possible; however, it was close to 100% at the beginning of accumulations based on propionic acid alone (Experiment set 2). It has been demonstrated that keeping an optimal active biomass specific growth rate enhances PHBV copolymer synthesis in *Cupriavidus necator* [36]. However, at high specific cell growth rates, more substrate is used for active biomass formation and less is available for PHA production. The specific cell growth rate in the present study was relatively low compared to pure cell cultures, and it was found that the higher the specific growth rate, the higher the %3HV (fitted data for specific growth rate and monomer specific synthesis rates are in the Supplementary Materials). Grousseau et al. [6] found that a higher PHB yield on substrate was obtained when the Entner–Doudoroff pathway was active. This pathway produces NADPH and is linked to anabolic requirements. It supports the idea that maintaining cellular growth offers an alternative pathway to TCA cycle for NADPH generation and it favours PHA synthesis.

When cells experienced a degree of cell growth limitation, a larger proportion of 3HB monomer units compared to 3HV monomers units was produced. In those cultures fed continuously with acetic acid (Experiment sets 1 and 3), reductive equivalents can be directly generated by acetyl-CoA pathway through the TCA cycle, which favors PHA production. Similar PHA fluxes were obtained using acetic or propionic acid as single substrates (Figure 4). However, total PHA yield on substrate was higher for cultures fed exclusively with acetic acid than cultures fed with propionic acid as sole substrate (Table 2). However, compared with accumulations fed with propionic acid exclusively (Experiment set 2) or periodically (Experiment set 4), acetic acid as a feed did not stimulate concurrent active growth and storage as much as propionic acid. Previous MFA studies have suggested that when acetic and propionic acids are fed simultaneously, the catabolic activity (TCA) primarily depends on acetic acid uptake [4]. According to the metabolic model, active biomass synthesis has a higher demand of ATP and NADH when it is generated from acetic acid rather than propionic acid. In further MFA calculations, the rate of active biomass synthesis is not considered as being proportional to the consumption rate of acetic and propionic acids. This has resulted in a non-redundant MFA system, which showed that when acetic acid and propionic acids are fed simultaneously, most of the new active biomass is synthesized from propionic acid uptake. Future studies where more experimental rates are available (such as CO_2_ evolution) would be required to test this hypothesis.

## 4. Conclusions

This study presented an analysis of PHA accumulation processes by mixed cultures when adopting different feeding strategies which favor concurrent cellular growth and carbon storage. Although higher maximum specific growth rates were achieved in cultures fed continuously with acetic acid as a sole substrate or as part of a mixture, constant cell growth was not achieved. Significant changes through time to the instantaneous 3HV content were observed under most accumulation conditions, and such changes cannot be adequately described by existing metabolic models. An alternating feeding strategy resulted in constant instantaneous 3HV content, despite the decarboxylation rate increasing with time. Overall, the 3HV monomer production rate is high. Finally, the incorporation of cellular metabolism in the evaluation of process performance for PHA production by mixed cultures offers an opportunity to help understand PHA polymer composition fluctuations and the carbon flux distribution in different cell physiological states. In this way, metabolic models can help improve the estimation of process final concentrations and yields. However, a better description of the 3HB:3HV fluctuations relative to active biomass growth needs a more detailed metabolic network to take into account the reactions in which NADPH and NADH are formed.

## Figures and Tables

**Table 1:** Experimental conditions in the PHA (Polyhydroxyalkanoates) fed-batch accumulations.

Experiment set	Substrate Composition and Feeding Strategy (gCOD Basis)	Experiment Label	Process Time (h)	Initial VSS (g·L^−1^)	Total substrate added (gCOD)		Feed Concentration (gCOD·L^−1^)	Total Number of Pulses	
					HAc	HPr		HAc	HPr
1	100% Acetic acid	Exp 1	23.2	1.2	1374	-	96	138	-
		Exp 1′	21.9	1.4	1684	-	98	147	-
2	100% Propionic acid	Exp 2	23.3	1.2	-	939	106	-	77
		Exp 2′	24.6	1.7	-	1439	102	-	137
3	50% Acetic/50% propionic acid	Exp 3	24.2	1.5	600	600	98	95	
		Exp 3′	20.4	1.9	817	817	98	140	
4	100% Acetic acid—100% propionic acid (alternating)	Exp 4	22.3	1.4	1429	982	101/103	106	105
		Exp 4′	22.5	1.7	570	552	94/97	57	56

**Table 2:** PHA accumulation yields and kinetic parameters.

Experiment Label	$f_{Ac}$ Consumed (mol HAc/mol VFA)	$f_{Pr}$ Consumed (mol HPr/mol VFA)	%PHA Plateau (gPHA/gVSS)	%3HV (mol 3HV/mol PHA) at 20 h	*Y_PHA/S_* (gCOD PHA/gCOD VFA)	*Y_X/S_* (gCOD *X*/gCOD VFA)	μ_max_ (h^−1^)	Final *X*/*X*_0_ (gCOD/gCOD)	$-q_{Smax}$ (Cmol VFA/Cmol *X*·h)	$q_{PHAmax}$ (Cmol PHA/Cmol *X*·h)
Exp 1	1.0	0	0.56 ± 0.04	0	0.48 ± 0.02	0.17 ± 0.03	0.27 ± 0.03	2.20	0.75	0.33
Exp 1′	1.0	0	0.48 ± 0.06	0	0.38 ± 0.04	0.15 ± 0.01	0.21 ± 0.02	2.28	0.80	0.36
Exp 2	0	1.0	0.40 ± 0.04	74	0.31 ± 0.03	0.16 ± 0.03	0.18 ± 0.02	2.13	0.33	0.11
Exp 2′	0	1.0	0.48 ± 0.03	80	0.40 ± 0.07	0.18 ± 0.03	0.13 ± 0.02	2.09	0.33	0.18
Exp 3	0.64	0.36	0.48 ± 0.06	40	0.39 ± 0.03	0.17 ± 0.05	0.35 ± 0.05	1.88	0.63	0.19
Exp 3′	0.64	0.36	0.52 ± 0.03	42	0.45 ± 0.03	0.12 ± 0.05	0.27 ± 0.05	1.75	0.40	0.19
Exp 4	0.72	0.28	0.59 ± 0.03	34	0.52 ± 0.03	0.18 ± 0.02	0.22 ± 0.02	3.08	0.37	0.23
Exp 4′	0.64	0.36	0.52 ± 0.06	36	0.49 ± 0.03	0.20 ± 0.02	0.19 ± 0.02	1.98	0.45	0.20

All data in table recorded as ± 95% confidence interval where possible.

**Table 3:** Propionyl-CoA decarboxylation fraction, respiratory quotient and energy dissipated estimated by MFA (with standard deviation in brackets).

Experiment Set	Elapsed Duration	%Conversion PrCoA to AcCoA (mmol/mmol)		RQ (Cmmol CO_2_/mol O_2_)		ATP Dissipated (molATP/Cmmol VFA Consumed)	
1	2 pulses	-	-	1.19	(0.03)	0.57	(0.45)
	2 h	-	-	1.16	(0.03)	0.87	(0.46)
	5 h	-	-	1.13	(0.03)	1.32	(0.46)
	8 h	-	-	1.10	(0.04)	1.71	(0.45)
	12 h	-	-	1.07	(0.03)	2.34	(0.24)
	20 h	-	-	1.05	(0.04)	3.11	(0.44)
2	2 pulses	0.49	(0.11)	0.79	(0.03)	1.83	(1.36)
	2 h	0.51	(0.10)	0.80	(0.02)	2.09	(1.19)
	5 h	0.55	(0.07)	0.81	(0.01)	2.50	(0.90)
	8 h	0.59	(0.05)	0.81	(0.01)	2.84	(0.67)
	12 h	0.65	(0.03)	0.82	(0.01)	3.21	(0.43)
	20 h	0.75	(0.01)	0.83	(0.01)	3.68	(0.08)
3	2 pulses	0.27	(0.04)	1.08	(0.06)	0.62	(0.38)
	2 h	0.39	(0.02)	1.01	(0.03)	1.17	(0.26)
	5 h	0.55	(0.02)	0.99	(0.02)	1.99	(0.18)
	8 h	0.69	(0.02)	0.95	(0.01)	2.66	(0.08)
	12 h	0.85	(0.03)	0.95	(0.00)	3.37	(0.04)
	20 h	0.97	(0.01)	1.01	(0.08)	0.46	(0.28)
4	2 pulses	0.44	(0.05)	1.14	(0.11)	0.27	(0.04)
	2 h	0.46	(0.01)	1.10	(0.10)	0.46	(0.19)
	5 h	0.48	(0.07)	1.05	(0.08)	0.76	(0.59)
	8 h	0.54	(0.07)	1.01	(0.05)	1.30	(0.49)
	12 h	0.60	(0.07)	0.99	(0.03)	1.83	(0.35)
	20 h	0.67	(0.05)	0.97	(0.01)	2.22	(0.68)

## Supplementary Materials

The following are available online at www.mdpi.com/2306-5354/4/1/20/s1, Figure S1: Experimental data for PHA fraction and relative active biomass production; (**a**) Experiment set 1: 100% acetic acid; (**b**) Experiment set 2: 100% propionic acid. Figure S2: Fitted data for specific growth rate and monomer specific synthesis rate. Experiment set 1: 100% acetic acid; Experiment set 2: 100% propionic acid; Experiment set 3: 50% acetic acid and 50% propionic acid fed simultaneously; Experiment set 4: 100% acetic acid alternating with 100% propionic acid, Figure S3: NADH generated and consumed at different stages of culture obtained by metabolic flux analysis (MFA). (**a**) Experiment set 1: 100% acetic acid; (**b**) Experiment set 2: 100% propionic acid, Figure S4: Internal carbon flux distribution (v3, v6, v9, v10, vX) normalised respect to substrate uptake rate. (**a**) Experiment set 1: 100% acetic acid; (**b**) Experiment set 2: 100% propionic acid; (**c**) Experiment set 3: 50% acetic acid and 50% propionic acid fed simultaneously; (**d**) Experiment set 4: 100% acetic acid alternating with 100% propionic acid, Table S1: Metabolic network for PHA processes by microbial mixed cultures used in the present work, Table S2: Microstructure of PHA copolymer samples by 13C NMR analysis.

## Abbreviations

%3HV	3HV fraction in total PHA (mol 3HV/mol PHA)
%3HV^inst^	Instantaneous 3HV content (mol 3HV/mol PHA)
%PHA	PHA intracellular content (gPHA/gVSS)
%PHA_0_	Initial PHA intracellular content (gPHA/gVSS)
$f_{Pr}$	Fraction of propionic acid uptake to total carbon uptake flux
3HB	3-Hydroxybutyrate
3HV	3-Hydroxyvalerate
AcCoA	Acetyl-CoA
ATP	Adenosine triphosphate
CO_2_	Carbon dioxide
CoA	Coenzyme A
COD	Chemical oxygen demand
*f_PHA_*	PHA fraction with respect to active biomass (PHA/X)
HAc	Acetic acid
HPr	Propionic acid
NAD^+^	Nicotinamide adenine dinucleotide (oxidised)
NADH	Nicotinamide adenine dinucleotide (reduced)
NADP^+^	Nicotinamide adenine dinucleotide phosphate (oxidised)
NADPH	Nicotinamide adenine dinucleotide phosphate (reduced)
PHA	Poly(3-hydroxyalkanoate) or PHA concentration at certain time
PHA_0_	Initial PHA concentration
PHB	Poly(3-hydroxybutyrate)
PHV	Poly(3-hydroxyvalerate)
PrCoA	Propionyl-CoA
*q_HAc_*	Acetic acid specific consumption rate (Cmol HAc/Cmol X·h)
*q_HB_*	Specific 3HB monomer synthesis rate (Cmol 3HB/Cmol X·h)
*q_HPr_*	Propionic acid specific consumption rate (Cmol HPr/Cmol X·h)
*q_HV_*	Specific 3HV monomer synthesis rate (Cmol 3HV/Cmol X·h)
*q_PHA_*	Specific PHA synthesis rate (Cmol PHA/Cmol X·h)
*q_S_*	Specific VFA consumption rate (Cmol VFA/Cmol X·h)
RQ	Respiratory quotient
TCA	Tricarboxylic acid cycle
*v*	Reaction rate (Cmol/Cmol X·h)
VFA	Volatile fatty acid
VSS	Volatile suspended solids
X	Active biomass
X_0_	Initial active biomass

## References

[1] Laycock B., Halley P., Pratt S., Werker A., Lant P. (2013). The chemomechanical properties of microbial polyhydroxyalkanoates. Prog. Polym. Sci..

[2] Filipe C.D.M., Daigger G.T., Grady C.P.L. (2001). A metabolic model for acetate uptake under anaerobic conditions by glycogen accumulating organisms: Stoichiometry, kinetics, and the effect of pH. Biotechnol. Bioeng..

[3] Taguchi K., Taguchi S., Sudesh K., Maehara A., Tsuge T., Doi Y. (2005). Metabolic pathways and engineering of polyhydroxyalkanoate biosynthesis. Biopolym. Online.

[4] Dias J.M.L., Oehmen A., Serafim L.S., Lemos P.C., Reis M.A.M., Oliveira R. (2008). Metabolic modelling of polyhydroxyalkanoate copolymers production by mixed microbial cultures. BMC Syst. Biol..

[5] Lemos P.C., Serafim L.S., Santos M.M., Reis M.A.M., Santos H. (2003). Metabolic pathway for propionate utilization by phosphorus-accumulating organisms in activated sludge: C-13 labeling and in vivo nuclear magnetic resonance. Appl. Environ. Microb..

[6] Grousseau E., Blanchet E., Deleris S., Albuquerque M.G.E., Paul E., Uribelarrea J.L. (2013). Impact of sustaining a controlled residual growth on polyhydroxybutyrate yield and production kinetics in *Cupriavidus necator*. Bioresour. Technol..

[7] Gurieff N. (2007). Production of Biodegradable Polyhydroxyalkanoate Polymers Using Advanced Biological Wastewater Treatment Process Technology. Ph.D. Thesis.

[8] Serafim L.S., Lemos P.C., Torres C., Reis M.A.M., Ramos A.M. (2008). The influence of process parameters on the characteristics of polyhydroxyalkanoates produced by mixed cultures. Macromol. Biosci..

[9] Albuquerque M.G.E., Martino V., Pollet E., Averous L., Reis M.A.M. (2011). Mixed culture polyhydroxyalkanoate (PHA) production from volatile fatty acid (VFA)-rich streams: Effect of substrate composition and feeding regime on pha productivity, composition and properties. J. Biotechnol..

[10] Ivanova G., Serafim L.S., Lemos P.C., Ramos A.M., Reis M.A.M., Cabrita E.J. (2009). Influence of feeding strategies of mixed microbial cultures on the chemical composition and microstructure of copolyesters p(3HB-*co*-3HV) analyzed by NMR and statistical analysis. Magn. Reson. Chem..

[11] Arcos-Hernandez M.V., Laycock B., Donose B.C., Pratt S., Halley P., Al-Luaibi S., Werker A., Lant P.A. (2013). Physicochemical and mechanical properties of mixed culture polyhydroxyalkanoate (PHBV). Eur. Polym. J..

[12] Johnson K., Van Loosdrecht M.C.M., Kleerebezem R. (2010). Influence of ammonium on the accumulation of polyhydroxybutyrate (PHB) in aerobic open mixed cultures. J. Biotechnol..

[13] Pardelha F., Albuquerque M.G.E., Reis M.A.M., Dias J.M.L., Oliveira R. (2012). Flux balance analysis of mixed microbial cultures: Application to the production of polyhydroxyalkanoates from complex mixtures of volatile fatty acids. J. Biotechnol..

[14] Serafim L.S., Lemos P.C., Oliveira R., Ramos A.M., Reis M.A.M. (2004). High storage of PHB by mixed microbial cultures under aerobic dynamic feeding conditions. Eur. Symp. Environ. Biotechnol..

[15] Valentino F., Karabegouic L., Majone M., Morgan-Sagastume F., Werker A. (2015). Polyhydroxyalkanoate (PHA) storage within a mixed-culture biomass with simultaneous growth as a function of accumulation substrate nitrogen and phosphorus levels. Water Res..

[16] Jiang Y., Hebly M., Kleerebezem R., Muyzer G., van Loosdrecht M.C.M. (2011). Metabolic modeling of mixed substrate uptake for polyhydroxyalkanoate (PHA) production. Water Res..

[17] Pardelha F., Albuquerque M.G.E., Reis M.A.M., Oliveira R., Dias J.M.L. (2014). Dynamic metabolic modelling of volatile fatty acids conversion to polyhydroxyalkanoates by a mixed microbial culture. New Biotechnol..

[18] Murugan Janarthanan O., Laycock B., Montano-Herrera L., Lu Y., Arcos-Hernandez M.V., Werker A., Pratt S. (2016). Fluxes in PHA-storing microbial communities during enrichment and biopolymer accumulation processes. New Biotechnol..

[19] Werker A.G., Bengtsson S.O.H., Karlsson C.A.B. (2011). Method for Accumulation of Polyhydroxyalkanoates in Biomass with on-Line Monitoring for Feed Rate Control and Process Termination.

[20] Morgan-Sagastume F., Pratt S., Karlsson A., Cirne D., Lant P., Werker A. (2011). Production of volatile fatty acids by fermentation of waste activated sludge pre-treated in full-scale thermal hydrolysis plants. Bioresour. Technol..

[21] American Public Health Association (APHA) (1995). Standard Methods for the Examination of Water and Wastewater.

[22] Motulsky H.J. (2007). Prism5 Statistics Guide.

[23] Zwietering M.H., Jongenburger I., Rombouts F.M., Vantriet K. (1990). Modeling of the bacterial growth curve. Appl. Environ. Microb..

[24] Third K.A., Newland M., Cord-Ruwisch R. (2003). The effect of dissolved oxygen on PHB accumulation in activated sludge cultures. Biotechnol. Bioeng..

[25] Villadsen J., Nielsen J., Lidén G. (2011). Bioreaction Engineering Principles.

[26] Klamt S., Saez-Rodriguez J., Gilles E.D. (2007). Structural and functional analysis of cellular networks with cellnetanalyzer. BMC Syst. Biol..

[27] Stephanopolulos G.N., Aristidou A.A., Nielsen J. (1998). Metabolic Engineering: Principles and Methodologies.

[28] Escapa I.F., Garcia J.L., Buhler B., Blank L.M., Prieto M.A. (2012). The polyhydroxyalkanoate metabolism controls carbon and energy spillage in *Pseudomonas putida*. Environ. Microbiol..

[29] Ren Q., de Roo G., Ruth K., Witholt B., Zinn M., Thony-Meyer L. (2009). Simultaneous accumulation and degradation of polyhydroxyalkanoates: Futile cycle or clever regulation?. Biomacromolecules.

[30] Arias S., Bassas-Galia M., Molinari G., Timmis K.N. (2013). Tight coupling of polymerization and depolymerization of polyhydroxyalkanoates ensures efficient management of carbon resources in *Pseudomonas putida*. Microb. Biotechnol..

[31] Lefebvre G., Rocher M., Braunegg G. (1997). Effects of low dissolved-oxygen concentrations on poly(3-hydroxybutyrate-*co*-3-hydroxyvalerate) production by *Alcaligenes eutrophus*. Appl. Environ. Microb..

[32] Lemos P.C., Serafim L.S., Reis M.A.M. (2006). Synthesis of polyhydroxyalkanoates from different short-chain fatty acids by mixed cultures submitted to aerobic dynamic feeding. J. Biotechnol..

[33] Yu J., Si Y.T. (2004). Metabolic carbon fluxes and biosynthesis of polyhydroxyalkanoates in *Ralstonia eutropha* on short chain fatty acids. Biotechnol. Prog..

[34] Kim J.I., Varner J.D., Ramkrishna D. (2008). A hybrid model of anaerobic *E. Coli* GJT001: Combination of elementary flux modes and cybernetic variables. Biotechnol. Prog..

[35] Zeng A.P., Ross A., Deckwer W.D. (1990). A method to estimate the efficiency of oxidative-phosphorylation and biomass yield from ATP of a facultative anaerobe in continuous culture. Biotechnol. Bioeng..

[36] Shimizu H., Kozaki Y., Kodama H., Shioya S. (1999). Maximum production strategy for biodegradable copolymer P(HB-*co*-HV) in fed-batch culture of *Alcaligenes eutrophus*. Biotechnol. Bioeng..

