# Supplementary material for: The Evolution of Polymer Composition during PHA Accumulation: The Significance of Reducing Equivalents
Source: Bioengineering (Basel). 2017 Mar 7;4(1):20. doi: 10.3390/bioengineering4010020 (PMC5590436; doi:10.3390/bioengineering4010020)
Supplement: Supplementary file 1 [file bioengineering-04-00020-s001.pdf]

Supplementary information A. Stoichiometry and PHA accumulation experimental results

Table S1. Metabolic network for PHA processes by microbial mixed cultures used in the present work (Pardelha et al. 2012)

	**Reactions**	HAc	HPr	AcCoA	PrCoA	O_2	NH_3	ATP	NADH	CO_2	AcCoA*	PrCoA*	X	3HB	3HV	**Flux**
$\mathbf{R_1}$	Acetic acid uptake	-1	-	1	-	-	-	-1	-	-	-	-	-	-	-	v_{HAc}
$\mathbf{R_2}$	Propionic acid uptake	-	-1	-	1	-	-	-0.67	-	-	-	-	-	-	-	v_{HPr}
$\mathbf{R_3}$	Propionyl-CoA Decarboxylation	-	-	1	-1.5	-	-	-	1.5	0.5	-	-	-	-	-	v_3
$\mathbf{R_4}$	Active biomass from Acetyl-CoA	-	-	-1.27	-	-	-0.2	-1.7	0.53	0.27	-	-	1	-	-	v_4
$\mathbf{R_5}$	Active biomass from Propioyl-CoA	-	-	-	-1.06	-	-0.2	-1.38	0.47	0.06	-	-	1	-	-	v_5
$\mathbf{R_6}$	TCA	-	-	-1	-	-	-	0.5	2	1	-	-	-	-	-	v_6
$\mathbf{R_7}$	Maintenance	-	-	-	-	-	-	-1	-	-	-	-	-	-	-	v_7
$\mathbf{R_8}$	Oxidative phosphorylation	-	-	-	-	-0.5	-	δ	-1	-	-	-	-	-	-	v_8
$\mathbf{R_9}$	Acetyl-CoA* formation	-	-	-1	-	-	-	-	-0.25	-	1	-	-	-	-	v_9
$\mathbf{R_{10}}$	Propionyl-CoA* formation	-	-	-	-1	-	-	-	-0.167	-	-	1	-	-	-	v_{10}
$\mathbf{R_{HB}}$	3HB monomer formation	-	-	-	-	-	-	-	-	-	-1	-		1	-	v_{HB}
$\mathbf{R_{HV}}$	3HV monomer formation	-	-	-	-	-	-	-	-	-	-0.4	-0.6		-	1	v_{HV}

δ – Oxidative phosphorylation efficiency; HAc – acetic acid; HPr – propionic acid; AcCoA – acetyl-CoA; PrCoA – propionyl-CoA; O_2 – oxygen; NH_3 – ammonium; ATP – adenosine triphosphate; NADH – nicotinamide adenine dinucleotide (reduced); CO_2 – carbon dioxide; AcCoA – reduced form of acetyl-CoA; PrCoA* – reduced form of propionyl-CoA; X – active biomass. The biomass formula is assumed $CH_{1.4}O_{0.4}N_{0.2}$. All stoichiometric coefficients are given in Cmmol*

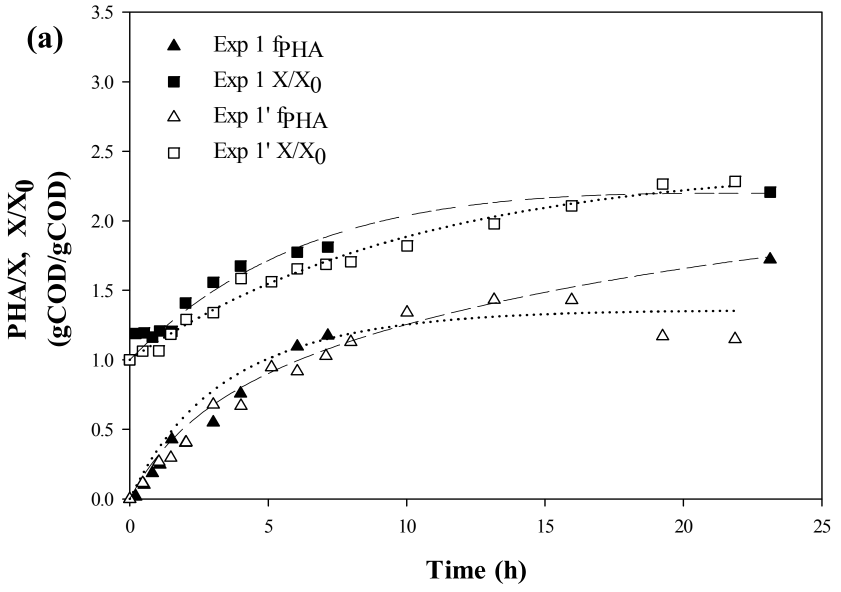

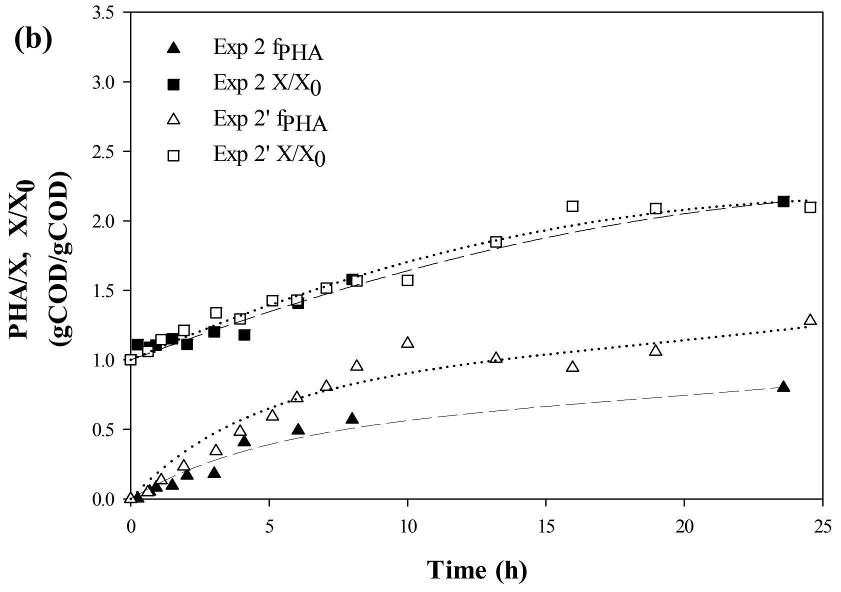

Figure S1. Experimental data for PHA fraction and relative active biomass production; (a) Experiment set 1: 100% acetic acid; (b) Experiment set 2: 100% propionic acid. ▲,△ – PHA fraction respect to active biomass concentration f_{PHA} (PHA/X). ■,□ – Active biomass concentration respect to the initial biomass concentration (X/X_0). Dotted lines represent fitted data. Coefficients are given on gCOD basis.

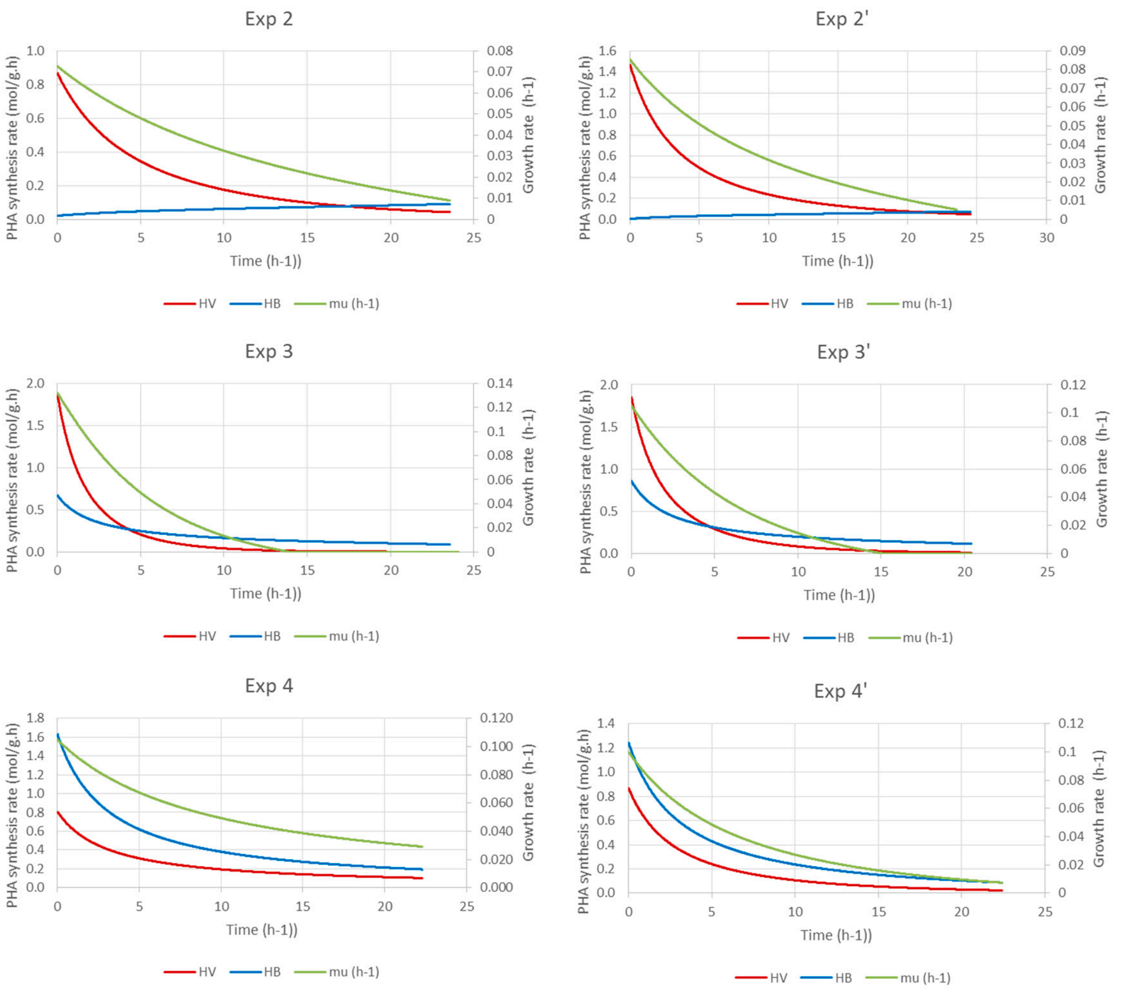

Figure S2. Fitted data for specific growth rate and monomer specific synthesis rate. Experiment set 1: 100% acetic acid; Experiment set 2: 100% propionic acid; Experiment set 3: 50% acetic acid and 50% propionic acid fed simultaneously; Experiment set 4: 100% acetic acid alternating with 100% propionic acid.

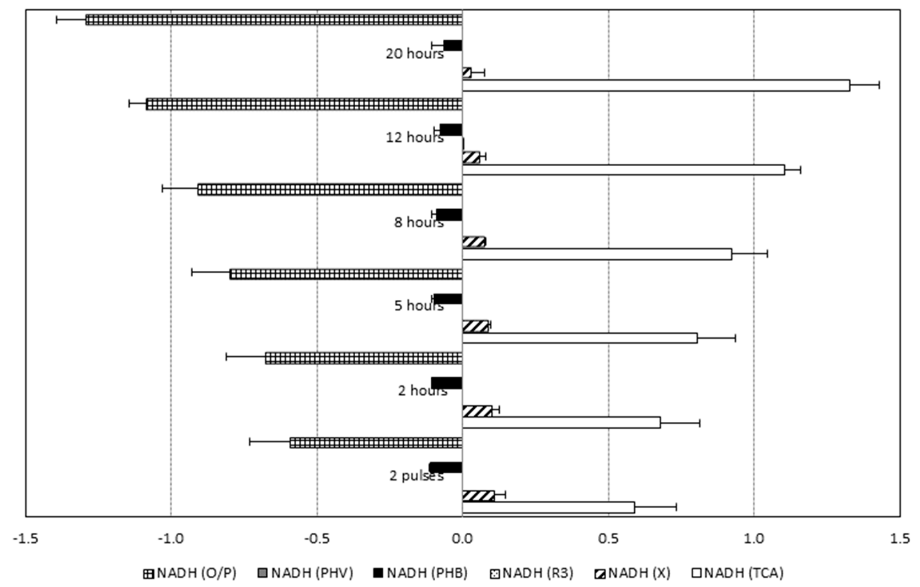

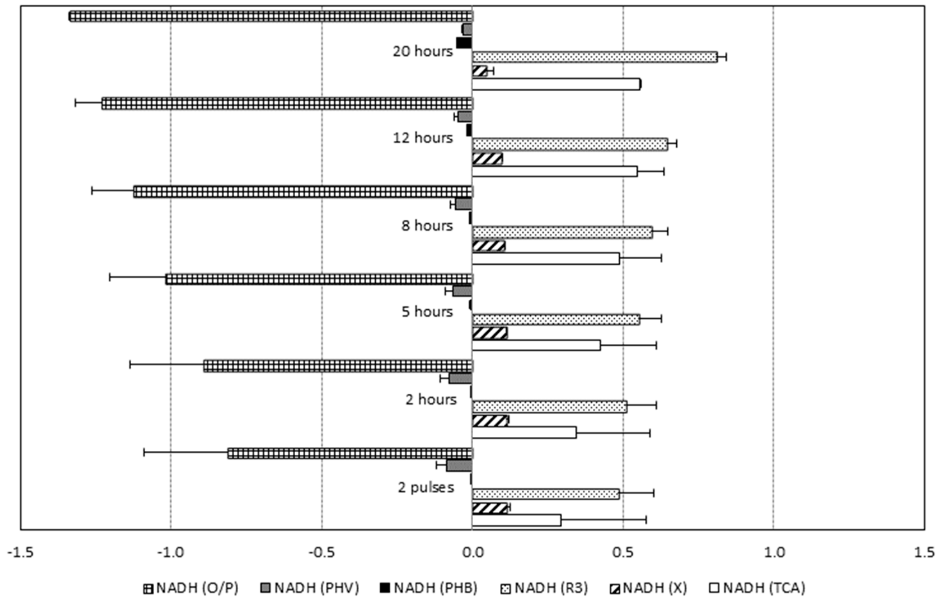

Figure S3. NADH generated and consumed at different stages of culture obtained by metabolic flux analysis (MFA). (a) Experiment set 1: 100% acetic acid; (b) Experiment set 2: 100% propionic acid.

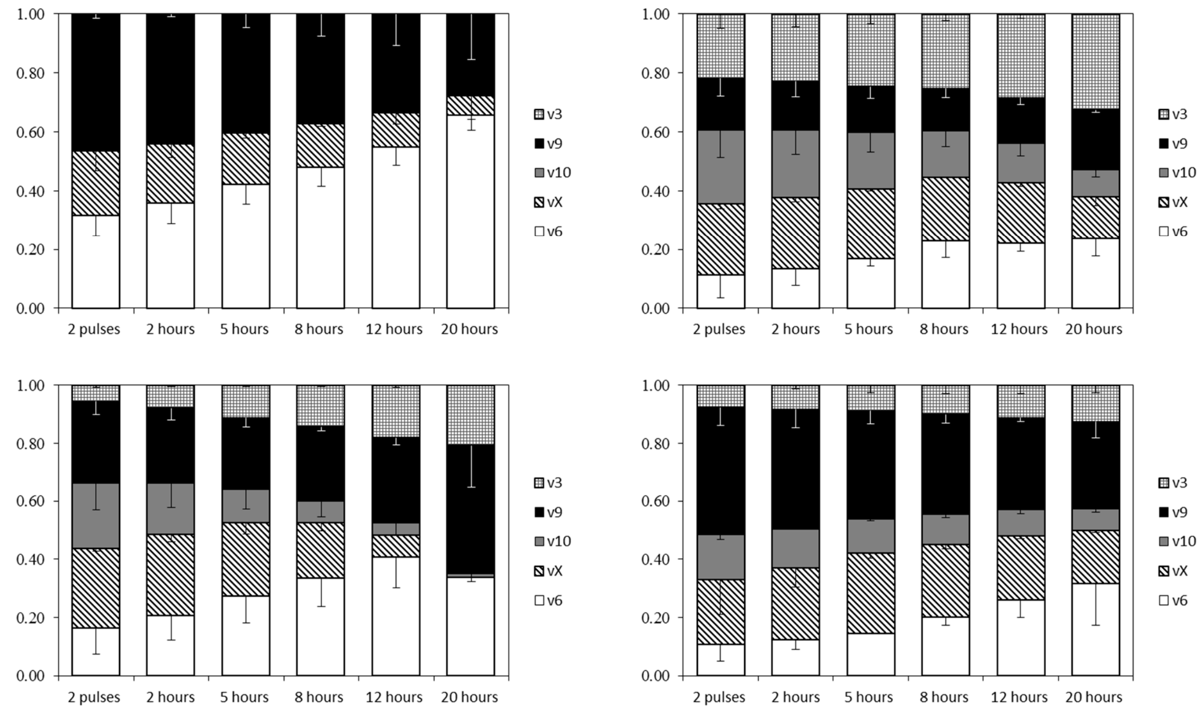

Figure S4. Internal carbon flux distribution (v_3, v_6, v_9, v_{10}, v_X) normalised respect to substrate uptake rate. (a) Experiment set 1: 100% acetic acid; (b) Experiment set 2: 100% propionic acid; (c) Experiment Set 3: 50% acetic acid and 50% propionic acid fed simultaneously; (d) Experiment set 4: 100% acetic acid alternating with 100% propionic acid.

Supplementary information B. Determination of microstructure of PHA samples

For the materials produced under alternating feeding strategy (materials 4, 4′ and 5), it was expected that acetic and propionic acid feeding pulses would produce repeated blocks of PHB and PHV rich sections, respectively. The polymer microstructure from the five experimental conditions was examined by ^{13}C NMR and the parameters *D* and *R* were calculated (Table S2).

Table S2. Microstructure of PHA copolymer samples by ^{13}C NMR analysis

Sample	Substrate	3HV (mol%)	*k*	*D*	*R*	L(EV)	L(VR)
**Exp 2**	100% Propionic acid	0.80	0.25	1.50	-	1.44	5.07
**Exp 2'**		n.d.	n.d.	n.d.	n.d.	n.d.	n.d.
**Exp 3**	50% Acetic/ 50% propionic acid	0.34	1.96	1.59	0.78	1.94	1.51
**Exp 3′**		0.35	1.87	1.86	0.70	2.30	1.53
**Exp 4**	100% Acetic acid – 100% propionic acid (alternating)	0.41	1.43	51.9	0.80	2.12	1.70
**Exp 4'**		0.31	2.24	3.75	0.50	2.89	1.45

Experimental number average sequence lengths of HV units – L (EV) and number average sequence length of randomly distributed HV units in copolymer – L(RV); R=L(RV)/L(EV). Ratio between concentration of 3HB and 3HV units – k.
n.d. not determined

Samples exhibited a D value equal or greater to 1.5, which indicates that in all cases, mixtures of copolymers and/or blocky copolymers were obtained. Higher *D* values were obtained for materials 4 and 4' when compared with materials obtained in accumulations fed with propionic only or a mixture of acetic and propionic acids (2, 3 and 3′). In the case of material 5, where larger feeding pulses were supplied, the *D* value was smaller than for materials 4 and 4'. A

particularly high *D* value (*D* = 51.9), indicating a probable block *co*-polymer, was obtained for material 4; this outcome is consistent with the results achieved by Arcos-Hernández et al. (2013). However, the switching feeding strategy in this work was based on alternating pulses while Arcos-Hernández et al. (2013) strategy was based on alternating feeds with a fixed timing. On the other hand, *R* values were above 0.70 for all materials, except for material 4′ for which *R* was 0.5.
